# Development and validation of a model that includes two ultrasound parameters and the plasma D-dimer level for predicting malignancy in adnexal masses: an observational study

**DOI:** 10.1186/s12885-019-5629-x

**Published:** 2019-06-11

**Authors:** Maciej Stukan, Michał Badocha, Karol Ratajczak

**Affiliations:** 1 Department of Gynecologic Oncology, Gdynia Oncology Center, Pomeranian Hospitals, Gdynia, Poland, Postal address: ul. Powstania Styczniowego 1, 81-519 Gdynia, Poland; 2 Department of Physical Chemistry, Gdańsk University of Technology, Gdańsk, Poland, Postal address: ul. Gabriela Narutowicza 11/12, 80-233 Gdańsk, Poland; 3 Karol Ratajczak Consulting, ul. Damroki 1A, 80-175, Gdańsk, Poland

**Keywords:** Ovarian cancer, Ultrasound, D-dimer, Calculation, Diagnosis, differential, Sensitivity and specificity

## Abstract

**Background:**

Pre-operative discrimination of malignant from benign adnexal masses is crucial for planning additional imaging, preparation, surgery and postoperative care. This study aimed to define key ultrasound and clinical variables and develop a predictive model for calculating preoperative ovarian tumor malignancy risk in a gynecologic oncology referral center. We compared our model to a subjective ultrasound assessment (SUA) method and previously described models.

**Methods:**

This prospective, single-center observational study included consecutive patients. We collected systematic ultrasound and clinical data, including cancer antigen 125, D-dimer (DD) levels and platelet count. Histological examinations served as the reference standard. We performed univariate and multivariate regressions, and Bayesian information criterion (BIC) to assess the optimal model. Data were split into 2 subsets: training, for model development (190 observations) and testing, for model validation (*n* = 100).

**Results:**

Among 290 patients, 52% had malignant disease, including epithelial ovarian cancer (72.8%), metastatic disease (14.5%), borderline tumors (6.6%), and non-epithelial malignancies (4.6%). Significant variables were included into a multivariate analysis. The optimal model, included three independent factors: solid areas, the color score, and the DD level. Malignant and benign lesions had mean DD values of 2.837 and 0.354 μg/ml, respectively. We transformed established formulae into a web-based calculator (http://gin-onc-calculators.com/gynonc.php) for calculating the adnexal mass malignancy risk. The areas under the curve (AUCs) for models compared in the testing set were: our model (0.977), Simple Rules risk calculation (0.976), Assessment of Different NEoplasias in the adneXa (ADNEX) (0.972), Logistic Regression 2 (LR2) (0.969), Risk of Malignancy Index (RMI) 4 (0.932), SUA (0.930), and RMI3 (0.912).

**Conclusions:**

Two simple ultrasound predictors and the DD level (also included in a mathematical model), when used by gynecologist oncologist, discriminated malignant from benign ovarian lesions as well or better than other more complex models and the SUA method. These parameters (and the model) may be clinically useful for planning adequate management in the cancer center. The model needs substantial validation.

**Electronic supplementary material:**

The online version of this article (10.1186/s12885-019-5629-x) contains supplementary material, which is available to authorized users.

## Background

An ultrasound examination performed by an experienced sonologist is considered the best diagnostic method for discriminating malignant from benign ovarian lesions [1–3]. Alternatively, optimal differential diagnosis of pelvic masses can be performed with predictive models that incorporate clinical and ultrasound variables [4–7]. Patients with tumors suspected for malignancy should be referred to a gynecologist oncologist (GO), because those treated in a referral center undergo cytoreduction to microscopic disease more often, and thus achieve significantly better overall survival [8–10]. Ovarian lesions considered indeterminate on an initial sonography should receive a “second step” evaluation, e.g. the patient might be referred to a GO [11]. It means, that this consultant has to perform an accurate presurgical differential diagnosis of the pelvic mass to ensure patients are correctly assigned the optimal surgical approach and must plan additional imaging, surgical team, suitable operating time and postoperative care for individuals with epithelial ovarian cancer (EOC). In many institutions, ultrasound examination performed by non-radiologist – the GO, is considered a primary imaging modality for differential diagnosing of pathological masses in the pelvis and even for the staging [12–16].

All patients with active malignancies demonstrate some degree of coagulation activation, including activated thrombin and fibrin formation [17]. Elevated plasma D-dimer (DD) levels were considered a prognostic factor of poor overall survival, independent of venous thromboembolism (VTE) [18, 19], and a potential biomarker for the preoperative differentiation of benign versus malignant ovarian masses [20–22].

The majority of clinical activity of the GO should be devoted to the management of patients with a gynecological cancer, thus this consultant should be equipped with simple, clinically useful diagnostic tools or experience for discriminating malignant from benign ovarian tumors. Some GO relay on pelvis/abdominal computed tomography [23] or diffusion-weighted magnetic resonance [24] only, while others perform comprehensive ultrasound assessment of pelvic masses, followed by ultrasound scanning of the pelvis and abdomen to diagnose and define distant disease [12, 15, 25].

We aimed to define key ultrasound and clinical variables and if different from previous studies, than to develop and validate a predictive model for calculating preoperative ovarian tumor malignancy risk in a gynecologic oncology referral center. We aimed to use ultrasound parameters, that would be relatively simple, not confusing and easily reproduced. We compared our model to a subjective ultrasound assessment (SUA) method and previously described models. We hypothesized that our set of variables or multivariable predictive model would outperform the SUA and other existing models in predicting the malignancy of adnexal masses.

## Methods

This prospective, single-center, observational study included consecutive patients with ovarian tumors that underwent surgery within 60 days of the ultrasound examination in the Department of Gynecologic Oncology, in Gdynia Oncology Center, Poland. Exclusion criteria were: a prior bilateral oophorectomy, pregnancy, refusal to undergo ultrasonography.

### Index test

All patients underwent transvaginal and transabdominal sonography (both during the same examination), according to a standardized gray-scale and Doppler protocol, before surgery, with high-quality ultrasound equipment. When patients exhibited multiple adnexal masses, the statistical analysis included only the mass with the most complex ultrasound morphology. When all masses had similar ultrasound morphology, we included only the most easily accessible to ultrasound examination.

The ultrasound protocol provided data on: the largest tumor diameter, locularity (multilocular if ≥2 locules, yes = 1, no = 0), solid areas (yes = 1, if any papillation or a solid tumor as described elsewhere [26], no = 0, if none), the presence of ascites (fluid outside the pouch of Douglas, yes = 1, no = 0), pelvic or abdominal metastases (any detected with ultrasound, e.g. carcinomatosis, yes = 1, no = 0); data on intratumoral vascularization included the qualitative Color Score (CS), a standardized terminology described elsewhere [26] (1 = no, 2 = minimal, 3 = moderate, 4 = very strong blood flow), and quantitative evaluations of the pulsatility index (PI), resistance index (RI), and peak systolic velocity (PSV), in areas with the highest blood flow velocity [26, 27]. When no blood flow or only venous flow was detected, the RI, PI, and PSV values were arbitrarily assigned values of 1, 1.2, and 10, respectively [28]. Preoperative data included patient age, menopausal status (postmenopausal was defined as: more than 1 year of amenorrhea without a diagnosis of any endocrine disease that could influence menstrual cycles; receiving hormonal replacement therapy for menopausal symptoms; or ≥ 50 years of age, with a previous hysterectomy), and laboratory data: cancer antigen 125 (CA-125), platelet count (PLT), and plasma DD level. Age and laboratory data were modelled continuously - no threshold cut-off values were used. Blood tests were performed 1–14 days prior to surgery. The DD level was tested in citrate plasma. Briefly, venous blood was drawn from peripheral vessels, centrifuged, and the supernatant was diluted with a 0.11 M (3.2%) buffered solution of sodium citrate, where the component ratio was 9:1, respectively. The DD level was determined with immunoturbidometry, measured with an automatic coagulation analyser (STA R Evolution and STA Compact, Diagnostica STAGO) and a special kit of agents (i.e., latex particles coated with DD-specific murine monoclonal antibodies). We used second generation immunoradiometric assay kits for detecting CA-125 II (Roche Diagnostics). Kits included the OC125 antibody. Laboratory tests were performed as part of routine preoperative assessment.

The protocol was amended in December 2015. Subsequently, we collected additional ultrasound data, including the maximal diameter of the largest solid component, the number of papillary projections, and the presence/absence of more than 10 locules, acoustic shadows, blood flow in papillations, and an irregular cyst wall [26, 29].

Calculations of other predictive models were performed according to instructions provided elsewhere: the International Ovarian Analysis Group (IOTA): the Logistic Regression 2 (LR2) [30], Simple Rules (SR) [31], the Assessment of Different NEoplasias in the adneXa (ADNEX) [29], and the Simple Rules risk calculation (SRrisk) [32]; two versions of the risk-of-malignancy index (RMI): RMI3 [33] and RMI4 [34] (Additional file 1). All ADNEX model calculations included the CA-125 level.

### SUA

The SUA was based on interviews, clinical and ultrasound (grey-scale and color Doppler) examinations. The examiner rated the level of diagnostic confidence as: certainly malignant; probably malignant; uncertain, but likely malignant; uncertain, but likely benign; probably benign; and certainly benign.

According to our institutional practice, an ultrasound scan was performed by a GO as a routine preoperative assessment. The examiner had 8–13 years of experience in ultrasound scanning; was blinded to histological reference diagnosis. All variables were recorded prospectively in a database (Excel, Microsoft Corporation), before surgery, and they were not changed thereafter. CA-125 results could be available to the examiner at the time of the ultrasound performance. All model’s calculations were performed after the study concluded; thus, the models played no role in the decision-making process. No imaging for VTE diagnosis was performed based solely on the finding that the plasma DD level was elevated.

### Reference standard

The outcome was the histological diagnosis of the entire ovarian mass removed during surgery. Based on histology, tumors were classified according the World Health Organization classification of tumors [35]. The reference test assessor was blinded to pre-surgery test details, but not to the general clinical impression and the CA-125 level.

Patients with early EOC received complete surgical staging, and advanced disease was treated with maximum debulking. Patients with benign tumors received individualized surgical treatment.

The study protocol was approved by the local Ethics Committee at Medical Council in Gdansk, Poland (KB – 36/17). This paper was written according to the standards for reporting of diagnostic accuracy (STARD) initiative [36] and transparent reporting of a multivariable prediction model for individual prognosis or diagnosis (TRIPOD) (Additional file 2) [37].

### Statistical analyses

Statistical analysis was performed with R software v.3.4.3 [38]. Mann-Whitney U test was used to check if there were significant differences in distributions between benign and malignant groups. The model was developed with the learning group, based on the original protocol. The model was validated and compared to other predictive models with the independent testing group, based on the amended protocol. The train and test sets were derived using a chronological split.

Before modelling, we selected variables in two stages. First, we reduced number of potential predictors based on subjective matter knowledge [39] and aims of our study (to select simple and easily reproduced parameters). Second, a univariable logistic regression model was constructed for each selected variable in the database of the learning group. Variables that achieved statistical significance (*p* < 0.05) in the univariable analysis were subsequently used in the multivariate model. This model used an algorithm of stepwise regression with backward selection. At each step, we removed the non-significant variable that caused the greatest reduction in the Bayes information criterion (BIC). The values of Akaike and Bayes (AIC/BIC) information criteria were reported since both serve valuable source of information [40]. However, in case of any incompatibilities between AIC and BIC, value of BIC was taken into consideration as it generally performs better, without overfitting the model [41].

Calibration [39] of the predicted probability of malignancy calculated by the developed model was investigated using calibration curves and by the ratio between average predicted probability of malignancy and observed prevalence of malignancy [42]. Plots were obtained using function from rms package [43]. A ratio of *<* 1 or > 1 suggests general underprediction or overprediction of the risk, respectively. The calibration curves link the predicted risk with the observed outcome using a non-parametric logistic regression model based on local regression [44].

Sensitivity, specificity, positive predictive values (PPV), negative predictive values (NPV) and receiver operating characteristic (ROC) curves, and the areas under the curve (AUC) were used to compare the predictive efficacies of the different models and SUA. The cut-off 0.5 was used to calculate measures of the performance (sensitivity, specificity, PPV and NPV) for models. Predictive models that were based on logistic regression analysis (including our model and IOTA models: LR2, ADNEX, and SRrisk) were evaluated as the probability of malignancy with values that ranged from 0 to 1 (0–100%) – we did not use stratification into low or high risk, nor any predefined border values. RMI cut-off values and detailed description of all model’s calculations are presented in an Additional file 1. We used no threshold cut-off values for CA-125, DD, PLT values.

There were no indeterminate results of index test and other predictive models, but SR. The IOTA-SR was converted into a risk estimate, to create the SRrisk algorithm, based on definitions published previously [32]. When the SUA was compared to the reference test and other predictive models, the first three levels of confidence were taken as malignant, and the last three were taken as benign. There were no indeterminate reference test results. In the statistical analysis, all benign epithelial and non-epithelial tumors were considered benign, and all epithelial malignant, borderline malignant, and non-epithelial malignant tumors were considered malignant.

There were no any missing data for the index test, nor for the reference standard.

The intended sample size was not determined before launching the study. However, we conducted the event per variable calculation to present the issue of the sample size and number of variables. According to recommendations, the suggested event per variable value should be more than 5–10 [39, 45].

## Results

**Fig. 1:**
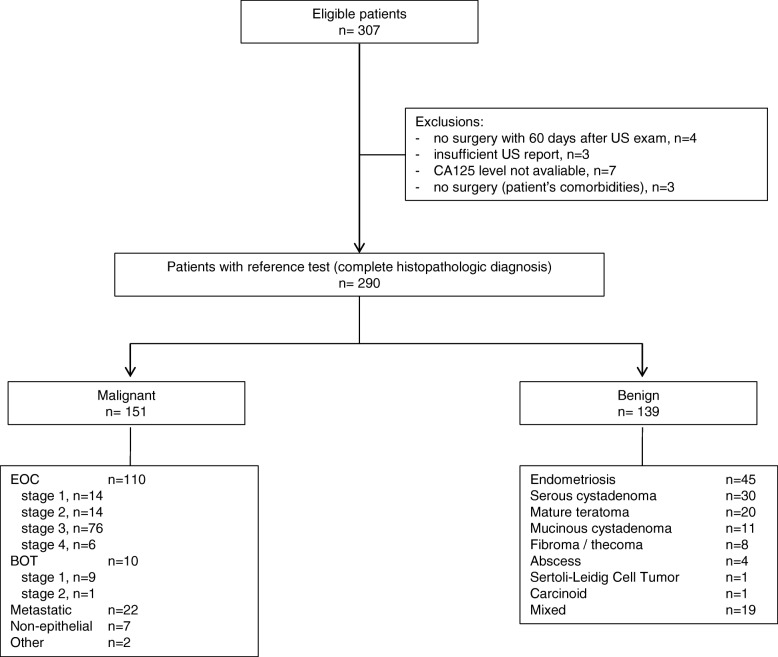
Flow diagram shows the inclusion and exclusion of eligible patients. *BOT* borderline malignant ovarian tumor, *CA-125* cancer antigen 125, *EOC* epithelial ovarian cancer, *US* ultrasound

From April 2012 to June 2017, we identified 307 eligible patients. Of these, 290 were included in the final analysis; 151 had malignant tumors (52%), and 139 had benign lesions (Fig. 1). The most common malignancy was EOC (72.8%), followed by metastatic cancer to the ovary (14.5%), borderline epithelial tumors (6.6%), non-epithelial malignancies (4.6%), and others (Fig. 1, Table 1).

**Table 1:** Histological types and subtypes of malignant disease

	*n* = 151	%
EOC, histology		
Serous	64	42.4
Endometrioid	15	9.9
Mucinous	8	5.3
Clearcell	8	5.3
Non-differentiated	6	4.0
Mixed	9	6.0
BOT, histology		
Serous	5	3.3
Mucinous	5	3.3
Non-epithelial, malignant		
Granulosa Cell Tumor, adult	3	2.0
Granulosa Cell Tumor, juvenile	1	0.7
Immature Teratoma	1	0.7
Mixed Germ Cell Tumor	1	0.7
Sertoli-Leidig Cell Tumor, G2	1	0.7
Metastatic, from:		
Large bowel	15	9.9
Stomach	2	1.3
Breast	1	0.7
Pancreas / biliary duct	1	0.7
Uterus, Cervix	1	0.7
Uterus, Endometrium	1	0.7
Lymphoma	1	0.7
Other, histology		
planoepithelial cancer	1	0.7
mesothelioma with ovarian involvement	1	0.7

*BOT* borderline malignant ovarian tumor, *EOC* epithelial ovarian cancer

Ovarian cancer, compared to benign disease, was associated with patient’s older age, post-menopause, and higher frequencies of the following tumor ultrasound features: the presence of solid areas, bilateral lesions, multilocularity, CS ≥2, the presence of ascites, and signs of metastases. Patients with malignancies had higher mean CA-125, plasma DD, and PLT count values than patients with benign lesions. The largest tumor diameters were similar between the benign and malignant groups (Table 2). Among patients with malignancies, the higher the stage, the higher the plasma DD level (*p* < 0.05) (Table 3). There were no differences in the median plasma DD level between patients with malignant epithelial primary (2.870, range, 0.138–28.618 μg/ml) and metastatic (2.828, range, 0.509–14.567 μg/ml) tumors.

**Table 2:** Patient characteristics and a comparison between patients with malignant and patients with benign ovarian lesions (*n* = 290)

Variable	All (*n* = 290)	Benign (*n* = 139)	Malignant (*n* = 151)	Mann-Whitney U test – *p* value
Age, years, median (Q1, Q3)	53 (41, 63)	45 (35, 55)	58 (49, 66)	*p* < 0.001
Postmenopausal, n(%)	158 (54.5)	48 (34.5)	110 (72.9)	*p* < 0.001
Ultrasound variables				
Mulilocular cyst, n(%)	131 (45.2)	46 (33.1)	85 (56.3)	*p* < 0.001
Solid areas, n(%)	199 (68.6)	52 (37.4)	147 (97.4)	*p* < 0.001
Bilateral lesions, n(%)	64 (22.1)	3 (2.26)	61 (40.4)	*p* < 0.001
Ascites, n(%)	57 (19.7)	3 (2.2)	54 (35.8)	*p* < 0.001
Metastases in abdominal cavity, n(%)	57 (19.7)	1 (0.7)	56 (37.1)	*p* < 0.001
Largerst diameter of tumor, mm, median (Q1, Q3)	67.0 (47.0, 122.8)	60.0 (50.0, 97.5)	84.0 (42.5, 140.0)	*p* = 0.15
Color Score	–	–	–	*p* < 0.001
Color Score 1, n(%)	178 (61.4)	117 (84.2)	61 (40.4)	
Color Score 2, n(%)	84 (29.0)	22 (15.8)	62 (41.1)	
Color Score 3, n(%)	25 (8.6)	0 (0.0)	25 (16.6)	
Color Score 4, n(%)	3 (1.0)	0 (0.0)	3 (2.0)	
RI, PI, PSV not detected, n(%)	212 (73.1)	121 (87.1)	91 (60.3)	*p* < 0.001
detected RI, median (range)	0.46 (0–0.73)	0.45 (0–0.73)	0.47 (0.20–0.70)	*p* = 0.506
detected PI, median (range)	0.70 (0–2.45)	0.73 (0–1.65)	0.66 (0.28–2.45)	*p* = 0.280
detected PSV, median (range)	14.3 (4.69–56.30)	14.40 (4.69–23.37)	14.14 (5.00–56.30)	*p* = 0.565
Laboratory variables				
CA125, U/ml, median (Q1, Q3)	75.5 (24.0, 438.0)	29.0 (15.0, 65.5)	291.0 (77.5, 897.0)	*p* < 0.001
PLT, G/l, median (Q1, Q3)	287.5 (239.0, 365.0)	262.0 (231.0, 302.5)	322.0 (252.0, 424.5)	*p* < 0.001
D-dimer, μg/ml, median (Q1, Q3)	0.779 (0.337, 3.039)	0.354 (0.277, 0.534)	2.837 (1.207, 6.064)	*p* < 0.001

*CA125* cancer antigen 125, *PLT* platelet count, *Q* quartile

**Table 3:** Plasma D-dimer mean levels for patients with primary ovarian cancer (including BOT) stratified by stage

Stage (FIGO 2014)	*N* = 129	Plasma D-dimer mean level [μg/ml]
I	26	1.816
II	18	2.490
IIIA1(ii)	5	1.445
IIIB	11	4.070
IIIC	63	7.609
IV	6	7.287

*BOT* borderline malignant ovarian tumor*, FIGO* International Federation of Gynecology and Obstetrics, One-way anova test for comparison of mean values in groups (*p* < 0.05)

The median time interval between the ultrasound examination and surgery was 1 day (range 0–60 days) for all included patients. No patient had clinical symptoms of VTE before surgery.

The learning group (*N* = 190, including 101 malignancies) was enrolled from April 2012 to May 2016. The testing group (*N* = 100, including 52 malignancies) was enrolled from June 2016 to June 2017. Data from both groups are presented in Additional file 3. The vascularization quantitative parameters (RI, PI, PSV) and the largest tumor diameter were excluded (learning group) before modelling based on subject matter knowledge [39] and lack of significant difference in univariate analysis, respectively. The following multivariate analysis was performed on learning group and included 11 clinical and ultrasound parameters from the protocol before amendment (Tables 4 and 5).

**Table 4:** Univariate and multivariate regression analysis for the learning group (n = 190)

	Univariate regression		Multivariate regression		
Variable	estimate	*p*-value	estimate (95% Cl)	OR	*p*-value
Age (years)	0.06938	< 0.001			
Menopausal status (postmenopausal)	0.8692	< 0.001			
Ultrasound parameters					
Mulilocular cyst	0.9457	0.002			
Solid areas	3.9671	< 0.001	3.7046 (1.9081, 5.5012)	40.6	< 0.001
Bilateral lesions	2.9750	< 0.001			
Ascites	2.8078	< 0.001			
Metastases in abdominal cavity	3.8865	< 0.001			
Largerst diameter of tumor [mm]	0.001807	0.45			
Color Score	1.6768	< 0.001	1.0313 (0.2119, 1.8508)	2.8	0.014
Laboratory variables					
CA125 [U/ml]	0.005822	< 0.001			
PLT [150–400 G/l]	0.007000	< 0.001			
D-dimer [μg/ml]	0.001967	< 0.001	0.0012 (0.0006, 0.0019)	1.0012	< 0.001
		intercept	−5.7496 (−7.8646, −3.6346)		< 0.001

*CA125* cancer antigen 125, *PLT* platelet count, *OR* odds ratio

**Table 5:** Model development: stepwise regression with backward selection and the Akaike and Bayes information criteria (AIC/BIC)

Steps	Model includes											AIC	BIC
	age	multilocular	SA	BL	ascites	metastases	MS	CA125	CS	PLT	DD		
0 - starting model	+	+	+	+	+	+	+	+	+	+	+	107.6212	149.8325
1 - “age” removed	–	+	+	+	+	+	+	+	+	+	+	105.8472	144.8115
2 - “metastases” removed	–	+	+	+	+	–	+	+	+	+	+	104.7247	140.4419
3 - “ascites” removed	–	+	+	+	–	–	+	+	+	+	+	103.1334	135.6037
4 - “mulilocular” removed	–	–	+	+	–	–	+	+	+	+	+	102.1436	131.3668
5 - “PLT” removed	–	–	+	+	–	–	+	+	+	–	+	101.5771	127.5533
6 - “MS” removed	–	–	+	+	–	–	–	+	+	–	+	102.1847	121.6669
7 - “CA125” removed	–	–	+	+	–	–	–	–	+	–	+	–	120.4145
8 - “BL” removed	–	–	+	–	–	–	–	–	+	–	+	–	119.9821
model: SA + CS	–	–	+	–	–	–	–	–	+	–	–	–	167.4295
model: SA + DD	–	–	+	–	–	–	–	–	–	–	+	–	121.9826
model: CS + DD	–	–	–	–	–	–	–	–	+	–	+	–	148.0912

*(+)*, included, *(−)* excluded, *BL* bilateral lesions, *CS* color score, *DD* D-dimer, *MS* menopausal status, *PLT* platelet count, *SA* solid areas

The analysis of the sample size and number of analyzed parameters revealed that the event per variable for the initial model was 8.1.

### The learning group, model development

The multivariate analysis results are shown in Tables 4 and 5. In Table 5 results of AIC are reported until values between AIC and BIC are inconsistent. The final, optimal model had the lowest BIC, AUC was 0.956 (CI:0.932–0.981), sensitivity 91.1% (83.9–95.2), specificity 85.4% (76.6–91.3), PPV 87.6% (80.0–92.6), NPV 89.4% (81.1–94.3), and included three independent factors. Malignancy probability was calculated according to the mathematical formula:

$$ \mathrm{Malignancy}\ \mathrm{probability}=\frac{1}{1+{e}^{-\left(-5.7495+3.7046\ast \mathrm{A}+1.0313\ast \mathrm{B}+1.2459\ast \mathrm{C}\right)}} $$

where *A, B* and *C* represented the solid areas, the CS, the plasma DD level (μg/ml), respectively.

For example, a patient with a solid area in the tumor (score 1), a CS-3 (score 3), and a plasma DD level of 2.293 μg/ml had a risk of malignancy 0.9803 (range: 0–1); that multiplied by 100% gave a risk of 98.03%. We designed an internet web-based tool (http://gin-onc-calculators.com/gynonc.php), to facilitate calculations.

### The testing group, model validation

**Fig. 2:**
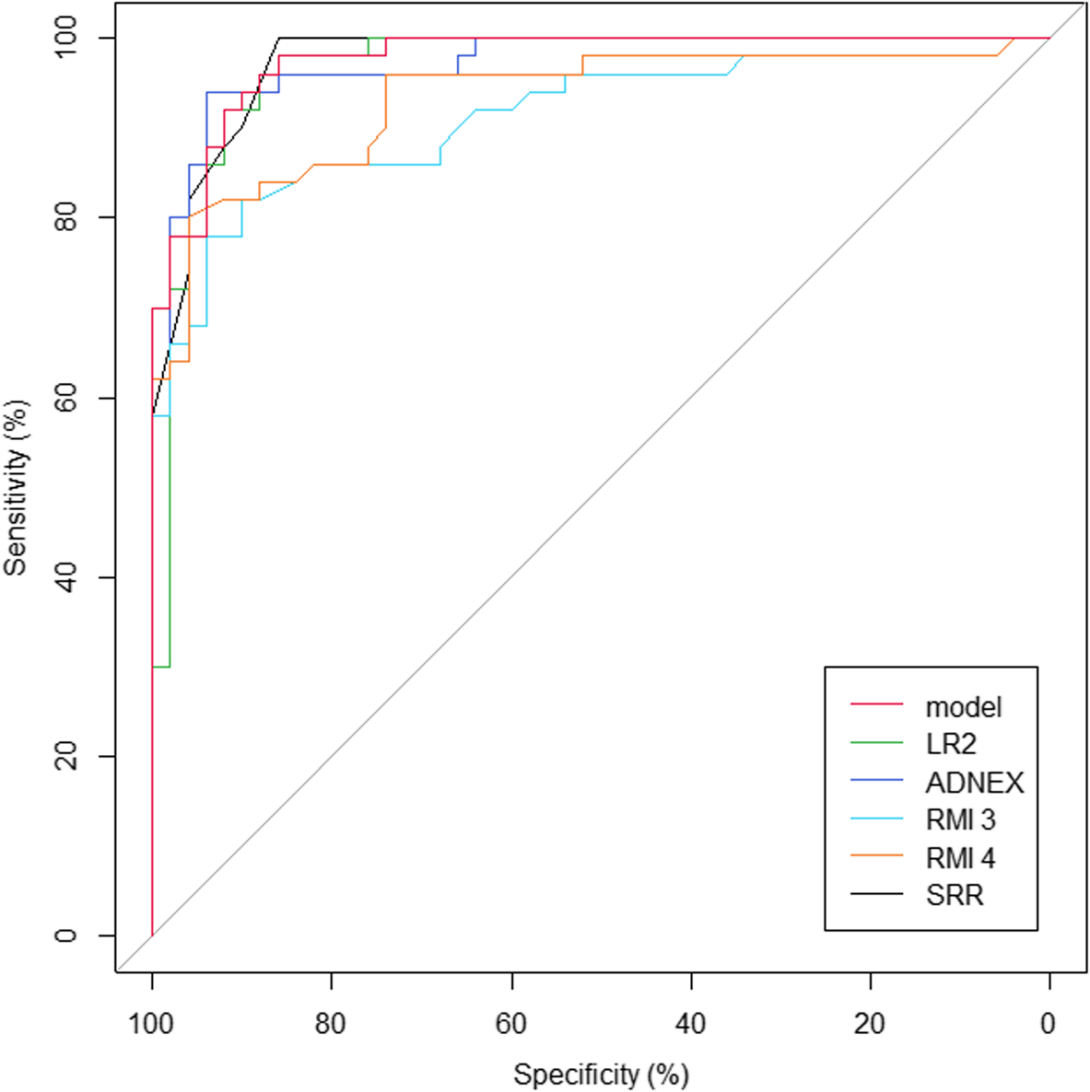
Receiver operating characteristic for the detection of malignant disease for different models. The data for the testing group (N = 100). Different line colors for different models *ADNEX, LR2, RMI3, RMI4, SRR -* abbreviations for models (details in the text), *model* the developed model

In the testing group, models with the highest AUC values were: our model, the SRrisk and the ADNEX. The calculated performance indices are shown in Table 6 and Fig. 2. The SUA showed high values overall. Our model had the highest sensitivity and NPV. The LR2 had the highest specificity and PPV, and the lowest sensitivity. Our model and the RMI4 had the lowest specificity and PPV. Among all malignant tumors, the SR classified 52% as malignant, 1.9% as benign, and 46.1% as inconclusive. Among all benign tumors, the SR classified 83.3% as benign, 4.2% as malignant, and 12.5%, as inconclusive. Among patients with malignancies, 46% of tumors were described as without a blood flow (B5), and only 4% had CS-4; there were no any other SR benign features. Among patients with benign tumors 8% had an irregular multilocular-solid tumor with largest diameter > 100 mm (M4), and there were no any other SR malignant features. Details are presented in Additional file 4.

**Table 6:** The calculated performance indices for different models and SUA for the testing group (*n* = 100)

Model / Method	Sensitivity (95% CI)	Specificity (95% CI)	PPV (95% CI)	NPV (95% CI)	AUC
ADNEX	88.0%	94.0%	93.6%	88.7%	0.972
	(76.2–94.4)	(83.8–97.9)	(82.8–97.8)	(77.4–94.7)	(0.946–0.999)
LR2	72.0%	98.0%	97.3%	77.8%	0.969
	(58.3–82.5)	(89.5–99.9)	(86.2–99.9)	(66.1–86.3)	(0.936–1.0)
RMI3	82.0%	88.0%	87.2%	83.0%	0.912
	(69.2–90.2)	(76.2–94.4)	(74.8–94.0)	(70.8–90.8)	(0.854–0.970)
RMI4	84.0%	86.0%	85.7%	84.3%	0.932
	(71.5–91.7)	(73.8–93.0)	(73.3–92.9)	(72.0–91.8)	(0.882–0.983)
SRrisk	82%	96.0%	95.3%	84.2%	0.976
	(69.2–90.2)	(86.5–98.9)	(84.5–98.7)	(72.6–91.5)	(0.953–0.999)
SUA	92.0%	94.0%	93.9%	92.2%	0.930
	(81.2–96.8)	(83.8–97.9)	(83.5–97.9)	(81.5–96.9)	(0.880–0.981)
Our model	96.0%	86.0%	87.3%	95.6%	0.977
	(86.5–98.9)	(73.8–93.0)	(76.0–93.7)	(85.2–98.8)	(0.955–0.999)

*95% CI* 95% confidence intervals, *ADNEX, LR2, RMI3, RMI4, SRrisk* abbreviations for different models (details in the text), *AUC* area under the curve, *NPV* negative predictive value, *PPV* positive predictive value, *RMI* risk of malignancy index (model), *SUA* subjective ultrasound assessment

Overall, our model slightly overpredicted the risk of malignancy – ratio of predicted and observed risk, 1.123 (Additional file 5).

## Discussion

This study identified two simple ultrasound predictors: solid areas and blood flow (described with the CS) in the tumor; and one clinical predictor: plasma DD level, that could discriminate malignant from benign adnexal masses. We incorporated these parameters into a predictive model (also as the web-based tool) for calculating the probability of malignancy. Our model performance was comparable to that of the more detailed and complex models: SRrisk, ADNEX and LR2 (IOTA), and better than of the RMI3 and RMI4 models. The SUA performed by a GO was also of high clinical value.

In a systematic review, based on studies published before March 2008, Geomini et al. concluded that the RMI was the model of choice for preoperative assessments of adnexal masses [4]. Later, Van Gorp et al. showed that the SUA was superior to the RMI [2]. Another review and meta-analysis of studies published in 2008–2013, showed that an evidence-based approach for preoperatively characterizing any adnexal mass should incorporate the IOTA-LR2, the SR or SUA [5]. Nunes et al. prospectively evaluated the IOTA-SR and performed a meta-analysis of studies (2008–2014) that utilized the model. They concluded that, in 76–89% of tumors, the SR protocol was accurate for diagnosing ovarian cancer. However, inconclusive cases required a “second- step” evaluation by an ultrasound expert [46]. Similarly, in a systematic review and meta-analysis of studies published in 1990–2015, Meys et al. also recommended the SR for a first evaluation of adnexal masses, and an expert SUA or the LR2 model for inconclusive cases [3]. In 2017, Westwood concluded that both the ADNEX and SR models offered higher sensitivity in assessing malignancy risk in adnexal masses than the RMI [7]. In contrast to some of those studies, we noted that about half of malignant tumors in the testing (validation) group (24/52) were classified as inconclusive and another half as malignant according to SR. These results may be attributable to high proportion of patients without blood flow detected in Doppler mode – the positive B5 feature, which marked together with at least one M feature gave the inconclusive SR results. Detailed search of patients (in testing group) with malignancies and inconclusive SR results (*n* = 24) revealed that there were 11 patients with primary peritoneal cancer (with only minimal ovarian involvement), for whom a set of features: B5 (no blood flow) and M1 (irregular solid) or M2 (ascites) was the most frequent. Other patients with malignancies and inconclusive SR results had either none of SR features present (*n* = 3), or had some of M features and the positive B5. Thus it seems, that in our setting, the most important feature that classified malignant tumors as inconclusive SR was the B5 (color score 1) added to at least one M feature. The Doppler scanning was always performed according to standardized technique. Interestingly, the CS was a powerful, independent variable in our multivariate analysis in the learning group, and later, in the testing group; the CS was important as a part of the model, that performed well as compared to others. Performance of SR for benign tumors was good, with 83% of tumors being correctly classified as benign,

Many predictive models have considered solid areas important in predicting malignancy risk in adnexal masses [27–29, 47–51]. Both solid/papillary structures and vascularization are important predictors in the SR, SRrisk and LR models [30–32]. The Consensus Recommendation markedly emphasized the importance of detecting the presence or absence of any solid/papillary structure in the tumor [11].

Many predictive models consider vascularization, defined with the CS or as blood flow in a papillary projection, an important variable [29–32, 51]. Quantitative parameters were also considered important [28, 51, 52], but some considered the Doppler to be negligible [53]. In our study, the CS was an independent predictive parameter. In contrast, RI, PI and PSV could be measured in only 40 and 13% of patients with malignant and benign tumors, respectively. Moreover, if measurable, there were no significant differences between both groups (Table 2). Consequently, these parameters were considered questionable in terms of clinical usefulness.

DD is a biomarker that globally indicates the activation of hemostasis and fibrinolysis. It is a degradation product of fibrin, which is produced when cross-linked fibrin is degraded by plasmin-induced fibrinolytic activity. As DD plasma levels are elevated after clot formation, the measurement of DD is routinely used in conjunction with clinical parameters in the initial assessment of suspected acute VTE [54]. Elevated DD levels may also be observed in other clinical settings, such as cancer, pregnancy and infectious diseases or following trauma and surgery [55]. Interestingly, a systemic activation of blood coagulation and procoagulant changes in the hemostatic system have frequently been observed in cancer patients, even in the absence of VTE. Moreover, coagulation activation, in particular thrombin generation and fibrin formation and dissolution, have been implicated in angiogenesis, tumor cell invasion, tumor progression, and metastatic spread. Tumor cells also possess strong procoagulant activities that induce local activation of the coagulation system and deposition of fibrin, which has an important role in the formation of tumor stroma and hematogenous spread of tumor cells [55]. DD is routinely not considered a cancer marker, however giving the fact of our results and observations discussed above and described elsewhere [55], the DD might appear as a biomarker for cancer.

The mean DD level was 0.71 μg/ml in the general population of patients with cancer [55] and 4.1–5.4 μg/ml among patients with EOC; in EOC, 90% of patients had DD levels over the cut-off value [18, 56, 57]. When patients with EOC underwent DD level measurements and imaging to detect VTE, the incidences of 16–25% for deep venous thrombosis, 0–11% for pulmonary thromboembolism, and 93–100% for asymptomatic disease was shown [18, 56, 57]. We noted that the mean DD level was elevated among patients with ovarian cancer (even in stage I) compared to patients with benign lesions. None of our patients had symptomatic VTE.

Some studies showed that with a cut-off value of 0.5 μg/ml, the DD level had 77–92% sensitivity, 72–94% specificity, 59–91% PPV, and 82–95% NPV for discriminating between benign and malignant ovarian lesions [20, 22]. The DD level combined with CA-125 (with a cut-off 65 U/ml) provided a sensitivity, specificity, PPV, and NPV of 73, 100, 100, and 81%, respectively [20]. The DD level seemed to outperform the CA-125 as a diagnostic biomarker, even for early stage EOC, where 73–83% of patients had elevated DD and 33–39% had elevated CA-125 [20, 22]. Interestingly, CA-125 in combinations with the red cell volume distribution width and the mean platelet volume (parameters usually measured as part of the whole blood cell count) may facilitate the early detection and differential diagnosis of ovarian cancer compared with benign ovarian tumors [58]. Measurement of serum CA-125 is routinely used to aid diagnosis and in a follow-up of patients with EOC. However, its utility to detect early disease is questionable [59]. Moreover, there is no overall survival advantage of early CA-125-directed retreatment for relapse [60]. RMI cannot be calculated without CA-125 [27]. The ADNEX model can be used with and without this variable [29]. It was shown that serum CA-125 may not be needed in models with a binary outcome (benign vs malignant) [61]. CA-125 is likely to be important for distinguishing between different types of malignant tumor [62]. In the ADNEX study it was shown that serum CA-125 level was important for good discrimination between stage II-IV cancer and stage I and secondary metastatic cancer. Deriving a similar model without CA-125 level as a predictor mainly affected discrimination between stage II-IV cancer and other malignancies: validation AUCs decreased from 0.82 to 0.59 (stage II-IV cancer *v* metastatic cancer), from 0.87 to 0.76 (stage II-IV cancer *v* stage I cancer), and from 0.95 to 0.91 (stage II-IV cancer *v* borderline tumors) [29]. Other models (e.g. SR, SRrisk, LR2) are based on ultrasound parameters only [30–32]. To our knowledge, this study was the first to show that, even in a multivariate analysis of various clinical and ultrasound variables, the DD level (not CA-125) was independently significant for differential diagnosing adnexal masses before surgery. Omission of CA-125 in our model with binary outcome (benign vs. malignant) was comparable to previous findings [61]. Also, other authors concluded that CA-125 did not add any diagnostic information when an ovarian mass was examined using ultrasound techniques by an experienced examiner [63]. Our model with DD level and without CA-125 had a comparable performance to ADNEX model calculated with CA-125.

This study had several strengths. It was performed prospectively, and it included consecutive, unselected patients diagnosed and treated in a regular center for gynecologic oncology. Additionally, the ultrasound was performed in a structured, planned manner, consistently for every patient. The index test was created with one set of patients (advanced statistical methods were used to select the most important variables), and it was validated with an independent set of patients, with a head-to-head comparison to results from other widely-used models, and to SUA. Other ultrasound-based models require many variables (6–12) to perform calculations. In contrast, our model was simpler, and it’s performance was comparable to other, more complex models. Moreover, we created a free-access, internet-based calculator to facilitate clinical use of our model.

This study also had some limitations. It was performed in a single institution, and the ultrasound was performed by a single examiner. Also, the patients had been referred to the gynecologic oncology department for surgery; thus, we did not include an observational arm. Our sample size was relatively small. IOTA models and the RMI were designed and validated on a larger number of patients and in many different clinical settings. Thus, our model lacked the generalizability of those models. However, our results could be useful for triaging patients at a referral center for gynecologic oncology. Another issue, is that there was no testing for VTE performed to compare it with DD levels. However, in order to test it, a comprehensive study should involve testing of every patient with Doppler ultrasound of lower extremities and pulmonary angio-computed tomography, and still would not cover testing for thrombosis in cancer tissue. A future study should involve a control group with adnexal mass and thrombosis, however this co-incidence might be incidental. There was no single patient who presented symptoms of VTE among all 290 included cases. One might note that an assessor of the reference test was not blinded to general clinical impression and CA-125 level, and consider this issue could lead to bias. However, this practice reflects clinical reality.

## Conclusions

We developed a model with two simple ultrasound predictors (solid areas and vascularization) and the plasma DD level for discriminating malignant from benign adnexal masses. This simple tool might be useful in referral centers for gynecologic oncology. The performance of our model was comparable to other, highly effective, though more complex models. Moreover, our model could be used to complement a subjective assessment. The model needs substantial validation.

## Additional files

Additional file 1: Definitions and mathematical formulae for calculation of different predictive models. (DOC 38 kb)

Additional file 2: TRIPOD checklist for the manuscript. (DOCX 90 kb)

Additional file 3: Clinical and ultrasound parameters in the learning (*N* = 190) and testing groups (*n* = 100). (DOC 61 kb)

Additional file 4: IOTA Simple Rules variables distribution in the testing group (*N* = 100) (malignant and benign groups compared with Fischer test). (DOCX 16 kb)

Additional file 5: Calibration curves of our model validation (n = 100). The grey line represents the perfect model used for comparison; black line (—) represents calibration plot; dotted line (∙∙∙∙) represents smooth fit of calibration plot using lowest method [41]. Predicted vs. observed risk 1.123. (PNG 7 kb)

## Abbreviations

ADNEX: Assessment of different NEoplasias in the adnexa (IOTA model)
AIC/BIC: Akaike and Bayes information criteria
AUC: Area under the curve
BOT: Borderline malignant ovarian tumor
CA-125: Cancer antigen 125
CS: Color Score
DD: D-dimer
EOC: Epithelial ovarian cancer
FIGO: International Federation of Gynecology and Obstetrics
GO: Gynecologist oncologist
IOTA: International Ovarian Analysis Group
LR2: Logistic Regression 2 (IOTA model)
NPV: Negative predictive value
PPV: Positive predictive value
RMI3: Risk of malignancy index (model modification 3)
RMI4: Risk of malignancy index (model modification 4)
SR: Simple Rules (IOTA model)
SRrisk: (SRR) Simple Rules risk calculation (IOTA model)
SUA: Subjective ultrasound assessment
VTE: Venous thromboembolism
