# Supplementary material for: Development and validation of a model that includes two ultrasound parameters and the plasma D-dimer level for predicting malignancy in adnexal masses: an observational study
Source: BMC Cancer. 2019 Jun 11;19:564. doi: 10.1186/s12885-019-5629-x (PMC6558858; doi:10.1186/s12885-019-5629-x)
Supplement: Supplementary file 1 — Definitions and mathematical formulae for calculation of different predictive models. (DOC 38 kb) [file 12885_2019_5629_MOESM1_ESM.doc]

**Additional file 1.**

**Definitions and mathematical formulae for calculation of different predictive models.**

The following models were calculated according to the definitions and mathematical formulae presented below:

**ADNEX** (IOTA) [29]

Detailed calculations of ADNEX model is described in Appendix D to the original publication [26], available online, open access. We used the smartphone applications provided by IOTA (<http://www.iotagroup.org/index.php/software>), using the discrimination benign vs. malignant.

**LR2** (IOTA) [30]

The model’s estimated probability of malignancy for an adnexal tumor equals 1*/(*1 + *e*−*z)*,

For LR2: *z* = −5*.*3718 + 0*.*0354*(*1*)* + 1*.*6159*(*2*)* + 1*.*1768*(*3*)* + 0*.*0697*(*4*)* + 0*.*9586*(*5*)* − 2*.*9486*(*6*)*

where:

(1) age of the patient (years);

(2) the presence of ascites (yes = 1, no = 0);

(3) the presence of blood flow within a papillary projection (yes = 1, no = 0);

(4) maximal diameter of the solid component (expressed in mm but with no increase above 50mm);

(5) irregular internal cyst walls (yes = 1, no = 0);

(6) the presence of acoustic shadows (yes = 1, no = 0).

We used the smartphone applications provided by IOTA: <http://www.iotagroup.org/index.php/software>

**Risk of malignancy (RMI):**

**RMI3** [33] = U x M x CA125; where U = the ultrasound score, M = menopausal status, and CA125 = the level of this marker. U was calculated as follows: multilocularity, solid areas, bilaterality, ascites and intraabdominal metastases each scored one point. A total ultrasound score of 0 or 1 yielded U = 1, and a score of ≥2 yielded U = 3. Premenopausal status yielded M = 1 and postmenopausal status yielded M = 3. The serum CA125 level was applied directly to the calculation [24, 30].

**RMI4** [34] = U x M x S x CA125, where U, M and CA125 were defined as above, and S was for a tumor largest diameter. A total ultrasound score of 0 or 1 yielded U = 1, and a score of ≥2 yielded U = 4. Premenopausal status yielded M = 1 and postmenopausal status yielded M = 4. A tumor size of <7 cm yielded S = 1, and ≥7 cm yielded S = 2. The serum level of CA125 was applied directly to the calculation [34].

For RMI 3 a cut-off was used to assign the lesion as benign (<200) and malignant (≥200) [27, 33]. For RMI 4 a cut-off level was 400 [34].

The RMI models calculations were performed with free-access web-based calculators: http://gin-onc-calculators.com/ovarian.php

**Simple Rules** (IOTA) [31]

*Rules for predicting a malignant tumor (M-rules)*

M1: Irregular solid tumor

M2: Presence of ascites

M3: At least four papillary structures

M4: Irregular multilocular solid tumor with largest diameter ≥100 mm

M5: Very strong blood flow (color score 4)

*Rules for predicting a benign tumor (B-rules)*

B1: Unilocular

B2: Presence of solid components where the largest solid component has a largest diameter *<*7mm

B3: Presence of acoustic shadows

B4: Smooth multilocular tumor with largest diameter *<*100 mm

B5: No blood flow (color score 1)

If one or more M-rules apply in the absence of a B-rule, the mass is classified as malignant.

If one or more B-rules apply in the absence of an M-rule, the mass is classified as benign.

If both M-rules and B-rules apply, the mass cannot be classified.

If no rule applies, the mass cannot be classified.

We used the smartphone applications provided by IOTA: <http://www.iotagroup.org/index.php/software>

**Simple Rules risk (SRrisk) calculation** (IOTA) [32]

Risk estimate is done using formula: exp(RS)/[(RS)], where:

- exp is the natural exponential function,

- RS is a regression score calculation according to formula:

RS = -0,97(intercept) - 3,41*B1 - 2,25*B2 - 1,66*B3 - 2,75*B4 - 1,86*B5 + 2,19*M1 + 2,65*M2 + 1,53*M3 + 0,98*M4 + 1,55*M5 + 0,92(ultrasound examination at oncology center)

where B1, B2, B3, B4, B5 and M1, M2, M3, M4, M5 are the same as described in Simple Rules above [31].
