# Supplementary material for: Development and validation of a model that includes two ultrasound parameters and the plasma D-dimer level for predicting malignancy in adnexal masses: an observational study
Source: BMC Cancer. 2019 Jun 11;19:564. doi: 10.1186/s12885-019-5629-x (PMC6558858; doi:10.1186/s12885-019-5629-x)
Supplement: Supplementary file 3 — Clinical and ultrasound parameters in the learning (N = 190) and testing groups (n = 100). (DOC 61 kb) [file 12885_2019_5629_MOESM3_ESM.doc]

Additional file 3. Clinical and ultrasound parameters in the learning (N=190) and testing groups (n=100).

| Variable | All (n=290) | Learning group (n=190) | Testing group (n=100) |
| --- | --- | --- | --- |
| Age, years, median (Q1, Q3) | 53 (41, 63) | 53 (42, 63) | 52 (40, 62) |
| Postmenopausal, n(%) | 158 (54.5) | 107 (56.3) | 51 (51.0) |
| Ultrasound variables |  |  |  |
| Mulilocular cyst, n(%) | 131 (45.2) | 85 (44.7) | 46 (46.0) |
| Solid areas, n(%) | 199 (68.6) | 125 (65.8) | 74 (74.0) |
| Bilateral lesions, n(%) | 64 (22.1) | 44 (23.2) | 20 (20.0) |
| Ascites, n(%) | 57 (19.7) | 40 (21.1) | 17 (17.0) |
| Metastases in abdominal cavity, n(%) | 57 (19.7) | 37 (19.5) | 20 (20.00) |
| Largerst diameter of tumor, median, mm (Q1, Q3) | 67.0 (47.0, 122.8) | 70.5 (47.0, 119.8) | 62.0 (47.5, 123.8) |
| Color Score | - | - | - |
| Color Score 1, n(%) | 178 (61.4) | 110 (57.9) | 68 (68.0) |
| Color Score 2, n(%) | 84 (29.0) | 59 (31.1) | 25 (25.0) |
| Color Score 3, n(%) | 25 (8.6) | 19 (10.0) | 6 (6.0) |
| Color Score 4, n(%) | 3 (1.0) | 2 (1.1) | 1 (1.0) |
| RI, not detected, n(%) | 212 (73.1) | 129 (67.9) | 83 (83.0) |
| PI, not detected, n(%) | 210 (72.4) | 127 (66.8) | 83 (83.0) |
| PSV, not detected, n(%)  RI, detected, median (range)  PI, detected, median (range)  PSV, detected, median (range) | 210 (72.4)  0.42 (0-0.73)  0.62 (0-2.45)  14.95 (4.69-56.30) | 127 (66.8)  0.47 (0-0.73)  0.72 (0-2.45)  13.80 (4.69-56.30) | 83 (83.0)  0.37 (0.21-0.66)  0.52 (0.28-1.13)  16.10 (7.64-40.50) |
| Laboratory variables |  |  |  |
| CA125, U/ml, median (Q1, Q3) | 75.5 (24.0, 438.0) | 81.0 (24.0, 419.5) | 65.0 (23.8, 446.5) |
| PLT, G/l, median (Q1, Q3) | 287.5 (239.0, 365.0) | 275.0 (230.0, 351.3) | 302.0 (257.5, 381.5) |
| D-dimer, µg/ml, median (Q1, Q3) | 0.779 (0.337, 3.039) | 0.735 (0.332, 2.835) | 0.917 (0.357, 3.342) |
| Histology, malignant, n(%) | 151 (52) | 101 (53.2) | 52 (52) |
|  |  |  |  |
| Ultrasound variables, after amendment | | | |
| Max. diameter of solid component, median, mm (range) |  |  | 42 (0 - 160) |
| More than 10 locules, n (%) |  |  | 11 (11) |
| Number of papillations, n (%) |  |  |  |
| 0, n (%) |  |  | 65 (65) |
| 1, n (%) |  |  | 14 (14) |
| 2, n (%) |  |  | 3 (3) |
| 3, n (%) |  |  | 5 (5) |
| >3, n (%) |  |  | 13 (13) |
| Acustic shadows, n (%) |  |  | 7 (7) |
| Blood flow in papillation, n (%) |  |  | 29 (29) |
| Irregular internal cyst wall, n (%) |  |  | 49 (49) |

*PI* pulsatility index, *PLT* platelet count, *PSV* peak systolic velocity, *Q* quartile, *RI* resistance index
