# Supplementary material for: Development and validation of a model that includes two ultrasound parameters and the plasma D-dimer level for predicting malignancy in adnexal masses: an observational study
Source: BMC Cancer. 2019 Jun 11;19:564. doi: 10.1186/s12885-019-5629-x (PMC6558858; doi:10.1186/s12885-019-5629-x)
Supplement: Supplementary file 4 — IOTA Simple Rules variables distribution in the testing group (N = 100) (malignant and benign groups compared with Fischer test). (DOCX 16 kb) [file 12885_2019_5629_MOESM4_ESM.docx]

Additional file 4. IOTA Simple Rules variables distribution in the testing group (N=100) (malignant and benign groups compared with Fischer test).

| Simple Rules variables | Malignant (histology)  n(%) | Benign (histology)  n(%) | p |
| --- | --- | --- | --- |
| B1 (unilocular cyst) | 0 (0) | 19 (39.58) | <0.001 |
|  |  |  |  |
| B2 (solid components present, but <7 mm) | 0 (0) | 7 (14.58) | 0.013 |
| B3 (acoustic shadows) | 0 (0) | 5 (10.42) | 0.057 |
|  |  |  |  |
| B4 (smooth multilocular tumor with largest diameter <100 mm) | 0 (0) | 9 (18.75) | 0.003 |
|  |  |  |  |
| B5 (no blood flow; color score 1) | 24 (46.15) | 42 (87.5) | 0.057 |
|  |  |  |  |
| M1 (irregular solid tumor) | 20 (38.46) | 0 (0) | <0.001 |
|  |  |  |  |
| M2 (ascites) | 18 (34.62) | 0 (0) | <0.001 |
|  |  |  |  |
| M3 (at least 4 papillary structures) | 14 (26.92) | 0 (0) | <0.001 |
|  |  |  |  |
| M4 (irregular multilocular-solid tumor with largest diameter >100 mm) | 27 (51.92) | 4 (8.33) | <0.001 |
|  |  |  |  |
| M5 (very strong flow; color score 4) | 2 (3.85) | 0 (0) | 0.497 |
|  |  |  |  |
| Benign | 1 (1.92) | 40 (83.33) | <0.001 |
|  |  |  |  |
| Malignant | 27 (52) | 2 (4.17) | <0.001 |
|  |  |  |  |
| Inconclusive | 24 (46.1) | 6 (12.5) | 0.006 |
|  |  |  |  |
| Total | 52(100) | 48(100) |  |
