# Supplementary figures and images for: Development and validation of a model that includes two ultrasound parameters and the plasma D-dimer level for predicting malignancy in adnexal masses: an observational study
Source: BMC Cancer. 2019 Jun 11;19:564. doi: 10.1186/s12885-019-5629-x (PMC6558858; doi:10.1186/s12885-019-5629-x)

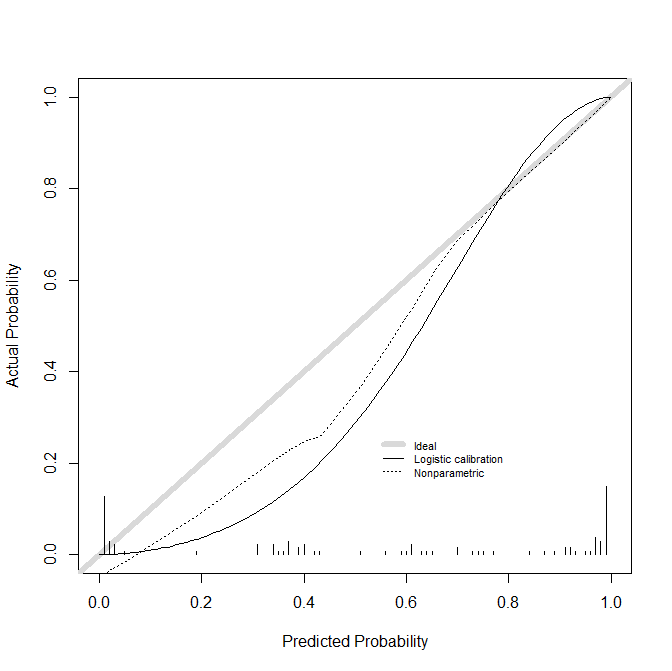

Supplement: Supplementary file 5 — Calibration curves of our model validation (n = 100). The grey line represents the perfect model used for comparison; black line (—) represents calibration plot; dotted line (∙∙∙∙) represents smooth fit of calibration plot using lowest method [41]. Predicted vs. observed risk 1.123. (PNG 7 kb) [file 12885_2019_5629_MOESM5_ESM.png]
